# Maternal and Perinatal Outcomes by Mode of Delivery in Senegal and Mali: A Cross-Sectional Epidemiological Survey

**DOI:** 10.1371/journal.pone.0047352

**Published:** 2012-10-08

**Authors:** Valérie Briand, Alexandre Dumont, Michal Abrahamowicz, Amadou Sow, Mamadou Traore, Patrick Rozenberg, Laurence Watier, Pierre Fournier

**Affiliations:** 1 Research Institute for Development, UMR 216, Paris, France; 2 UMR 216, Université Paris Descartes, Sorbonne Paris Cité, Paris, France; 3 Research Centre of CHU Sainte-Justine, Montreal, Canada; 4 Department of Epidemiology and Biostatistics, McGill University, Montreal, Canada; 5 Cabinet d'Etudes et de Recherche HYGEA, Dakar, Senegal; 6 Referral Health Center of the Commune V, Bamako, Mali; 7 Department of Obstetrics and Gynaecology, CHI Poissy Saint-Germain-en-Laye, Université de Versailles Saint-Quentin-en-Yvelines, Versailles, France; 8 U657, National Institute for Medical Research, Paris, France; 9 Pharmaco-Epidemiology and Infectious Diseases Unit, Pasteur Institute, Paris, France; 10 CRCHUM Research Centre, University of Montreal, Montreal, Canada; Tehran University of Medical Sciences, Islamic Republic of Iran

## Abstract

**Objective:**

In the context of rapid changes regarding practices related to delivery in Africa, we assessed maternal and perinatal adverse outcomes associated with the mode of delivery in 41 referral hospitals of Mali and Senegal.

**Study Design:**

Cross-sectional survey nested in a randomised cluster trial (1/10/2007–1/10/2008). The associations between intended mode of delivery and (i) in-hospital maternal mortality, (ii) maternal morbidity (transfusion or hysterectomy), (iii) stillbirth or neonatal death before Day 1 and (iv) neonatal death between 24 hours after birth and hospital discharge were examined. We excluded women with immediate life threatening maternal or fetal complication to avoid indication bias. The analyses were performed using hierarchical logistic mixed models with random intercept and were adjusted for women's, newborn's and hospitals' characteristics.

**Results:**

Among the 78,166 included women, 2.2% had a pre-labor cesarean section (CS) and 97.8% had a trial of labor. Among women with a trial of labor, 87.5% delivered vaginally and 12.5% had intrapartum CS. Pre-labor CS was associated with a marked reduction in the risk of stillbirth or neonatal death before Day 1 as compared with trial of labor (OR = 0.2 [0.16–0.36]), though we did not show that maternal mortality (OR = 0.3 [0.07–1.32]) and neonatal mortality after Day 1 (OR = 1.3 (0.66–2.72]) differed significantly between groups. Among women with trial of labor, intrapartum CS and operative vaginal delivery were associated with higher risks of maternal mortality and morbidity, and neonatal mortality after Day 1, as compared with spontaneous vaginal delivery.

**Conclusions:**

In referral hospitals of Mali and Senegal, pre-labor CS is a safe procedure although intrapartum CS and operative vaginal delivery are associated with increased risks in mothers and infants. Further research is needed to determine what aspects of obstetric care contribute to a delay in the provision of intrapartum interventions so that practices may be made safer when they are needed.

## Introduction

In high and middle-income countries, the reasons for performing a cesarean section (CS) – which is perceived as a safe procedure – have become much broader over time, leading to high and sometimes considerable increases in CS rates and decrease in spontaneous and instrumental vaginal delivery. However, CS may pose an intrinsic risk to the mother or the baby, independently of the women's medical conditions and obstetric complications, as shown in recent publications [1]–[4]. Few studies have assessed the intrinsic risk related to the different modes of delivery in low-income countries, where the deleterious effects of CS may be even higher due to delays in accessing referral health facilities [5], low safety of the procedure [6], and lack of human and material resources at the institutional level for managing emergency cases [7]. In the context of rapid changes regarding practices related to the mode of delivery in those countries – many of them reporting recent increases in CS rates [8], this issue is of upmost importance.

Herein, we have evaluated maternal, perinatal and neonatal outcomes by the mode of delivery using data collected from a large sample of women attending referral hospitals in Senegal and Mali.

## Materials and Methods

### Study site and Ethics statement

We conducted a cross-sectional epidemiological survey nested in a cluster-randomised trial (QUARITE trial) in Senegal and Mali (the QUARITE trial is registered on the Current Controlled Trials website under the number ISRCTN46950658 http://www.controlled-trials.com/). The trial was approved by the ethics committee of Sainte-Justine Hospital in Montreal, Canada, the “Comité National d'Ethique pour la Santé et les Sciences de la vie (CNESS)” of the Ministry of Health in Mali, and the “Conseil National de la Recherche en Santé (CNRS)” of the Ministry of Health in Senegal. Individual informed consent was not sought as clinical data were collected at the institutional level from medical records and hospital registers without identifying the individual women. Informed consent at the institutional level was obtained from the responsible authority (director of the centre and chief of maternity services) of the participating health facilities.

**Table 1:** Main reported indications for cesarean section (CS) according to the type of CS (n (%)) (Senegal and Mali, October 2007-October 2008).

		All CS (*n = *11,255)	Pre-labor CS (*n = *1,738)	Intrapartum CS (*n = *9,517)
Maternal indication		8,737 (77.6)		
	Prolonged/obstructed labor or suspected cephalopelvic disproportion	4,544 (40.4)	298 (17.2)	4,246 (44.6)
	Previous cesarean section	1,862 (16.6)	693 (39.9)	1,169 (12.3)
	Hypertensive disorders	535 (4.8)	82 (4.7)	453 (4.7)
	Abruptio placentae	658 (5.8)	0	658 (6.9)
	Other maternal indications$	1,138 (10.0)	477 (27.5)	661 (6.9)
Fetal indication		2,209 (19.6)		
	Fetal distress	1,808 (16.1)	18 (1.0)	1,790 (18.7)
	Other fetal indications$$	401 (3.5)	87 (5.0)	314 (3.3)
Not specified		309 (2.8)	65 (3.7)	244 (2.6)

$ Other maternal indications: post-term (*n = *124, 1.1%), maternal request (*n = *100, 0.9%), vaginal bleeding near full term (*n = *56, 0.5%), vaginal fistula (*n = *25, 0.2%), genital infection (*n = *6, 0.05%), HIV-infection (*n = *2), premature rupture of membranes (*n* = 1), CS performed for “other maternal indications” without any accurate diagnosis (*n = *824, 7.3%).

$$ Other fetal indications: suspected intrauterine growth retardation (*n = *20, 0.2%), breech presentation (*n = *4), CS performed for “other fetal indications” but without any accurate diagnosis (*n = *377, 3.3%).

The study protocol of the QUARITE trial has already been published [9]. Briefly, the trial aimed to assess the effectiveness of quality care improvement program (the Advances in Labor and Risk Management (ALARM) International Program) to reduce maternal mortality. The trial was conducted in 46 referral hospitals spread across both countries from October 2007 to September 2011. For the present study we used the data collected during the first year of the trial (from October 2007 to October 2008) while hospitals had not been randomised yet neither the ALARM International program implemented. This pre-intervention phase of the trial (October 2007-October 2008) aimed to provide baseline data to verify the comparability of the groups (ALARM program vs. control) in terms of the characteristics of the centres and of the women included. Five out of the 46 participating hospitals were excluded from our analysis because four did not carry out any CS during the study period (October 2007-October 2008) and one only had data from mid-2008. Among the 41 included hospitals (20 in Mali and 21 in Senegal), 12 were located in the capital, 14 were regional hospitals outside the capital and 15 were district hospitals.

### Data collection

Data was collected from medical records by trained midwives who were supervised by the national coordinators of the survey. In each country, data was collected on a daily basis on every woman who gave birth in every selected facility. The database for this study included information on maternal demographic characteristics, obstetric history (gravidity, parity and previous CS), prenatal care (number of antenatal care visits during the current pregnancy), management of labor and delivery, obstetric complications, and the vital status of both mother and child until hospital discharge. Medical conditions and obstetric complications were reported by midwives using open questions and a pre-defined list of diagnoses, except for pre-eclampsia/eclampsia, prolonged/obstructed labor, rupture of the uterus, haemorrhage and genital infection that must be reported systematically. To avoid under-reporting of in-hospital maternal mortality, a complementary procedure was carried out to identify the eligible maternal deaths among all the female deaths that occurred in the facility using the various registries available (admissions, hospitalizations, operating theatres and morgues).

For each institution, available equipment and human resources for obstetric care were recorded using a standardised inventory developed by Villar *et al.* for the WHO global survey on maternal and perinatal health [10]. Because resources may change over time, we collected the information at the beginning and at the end of the study period. Women who delivered during the first half time period of the study were assumed to have access to resources recorded by the first inventory. The second half time period corresponded to the second inventory. The scarcest resources were those related to the child (fetal monitoring, neonatal care, alpha fetoprotein and fetal pH scalp available in, respectively, 24.4%, 23.2%, 4.9% and 2.4% of hospitals). Other resources were available in 40.0% to 100.0% of hospitals such as blood bank (84.1%), safe blood (52.4%) and adult intensive care unit (41.5%). In all hospitals located in the capital there were obstetrics specialists and anaesthetists 24 h/day, in all regional hospitals there were obstetrics specialists, and anaesthetists 24 h/day were present in half of them. In contrast, in district hospitals, three-quarter had trained general practitioners only and anaesthetists on call.

### Study population and statistical analysis

All women with single pregnancy, living in Senegal or Mali, who delivered in the selected health facilities a newborn weighting more than 500 grams were eligible for analyses. To limit confounding by indication, referring to the fact that antenatal maternal morbidity may be both the indication for CS and the cause of maternal or perinatal death, we excluded women with immediate life threatening maternal or fetal complication (placenta praevia, severe pre-eclampsia, prerupture or rupture of the uterus, transverse lie, brow presentation, or major cephalopelvic disproportion [11]) (Figure S1). Most (98%) of these women had a cesarean section.

The two maternal outcomes were: (i) in-hospital maternal mortality defined as the death of the woman before hospital discharge and (ii) severe maternal morbidity, corresponding to blood transfusion or hysterectomy (the variable was coded 1 if transfusion and/or hysterectomy were reported and 0 otherwise). The two child outcomes consisted in (i) stillbirth or immediate neonatal mortality within 24 hours after birth (hereinafter referred to as “fetal/immediate neonatal mortality”), as we could not distinguish between ante- and intrapartum stillbirths and misclassifications may have occurred between fetal and neonatal death, and (ii) early neonatal mortality, which consisted in deaths that occurred more than 24 hours after birth and up to hospital discharge (hereinafter referred to as “neonatal mortality after Day 1”).

The primary predictor variable of interest was the mode of delivery. It was defined at four levels: spontaneous vaginal delivery, operative vaginal delivery (vacuum or forceps), emergency intrapartum CS (corresponding to CS indicated during either spontaneous or induced labor), and pre-labor CS (corresponding to CS scheduled before the onset of labor).

First, we based the analyses on the concept of “intention-to-treat” in comparing all women with intended pre-labor CS with all women who had a trial of labor, which could result in spontaneous or operative vaginal delivery or intrapartum CS. Then, among women with a trial of labor, we compared those with intrapartum CS with those who delivered vaginally: spontaneous and operative delivery (forceps and/or vacuum). Finally, we compared pre-labor CS with spontaneaous vaginal delivery.

For each outcome variable of interest, the analysis was performed using a two-step procedure. As the first step, we examined potential confounders of the association between specific maternal or child outcome and mode of delivery at individual level (woman or newborn characteristics). These variables were selected based on previous studies in low- or middle-income countries [1], [12]: age (2 classes: <35 years, ≥35 years), parity (2 classes: nulliparous, parous), number of antenatal care visits (2 classes: 0, ≥1), previous CS, medical conditions diagnosed before index pregnancy (2 classes: none versus at least one of the following conditions reported: HIV, chronic respiratory conditions, cardiac or renal diseases, sickle cell trait and chronic hypertension), referral from another health facility – which was considered as a potential marker for more severe conditions because of delays due to large travel distances or lack of transportation –, pregnancy-induced hypertension or mild pre-eclampsia, vaginal bleeding near full term, premature rupture of the membranes, chorioamniotis and other medical/obstetric conditions diagnosed during current pregnancy but before the onset of labor (2 classes: none versus at least one of the following conditions reported: pyelonephritis or urinary infection, chorioamniotis, severe maternal anaemia, malaria, gestational diabetes, suspected intrauterine death and suspected intrauterine growth retardation). We considered that women did not have a condition if it had not been reported by a midwife. Birth weight was always included in the models that were fitted to child outcomes (the variable was categorised in 6 classes: <2,000 grams, [2,000–2,500], [2,500–3,000], [3,000–3,500], [3,500–4,000], ≥4,000). First, tri-variate analyses (i.e., adjusted for country and time period) were performed to assess crude associations between mode of delivery and all the aforementioned individual-level variables. Then, a multivariable analysis was conducted. All variables were included in the final model, regardless of their associations with outcomes in tri-variate analyses.

As the second step of these analyses, we examined potential confounders, at institutional-level (hospital characteristics), of the associations between the mode of delivery and specific maternal or child outcome, while adjusting for individual factors that were selected into the multivariable model estimated at the first step. Institutional factors considered for these analyses were selected *a priori* based on essential resources for emergency obstetric care defined by Villar *et* *al*. for the WHO [10]. The final multivariable model included only those institutional variables that were selected by a forward-stepwise procedure (with a *P*<0.01 criterion for entry). We used a forward elimination procedure to account for very high sample size and high correlation between institutional variables. The time period and the country were forced into the final multivariable model.

Two sensitivity analyses were conducted. First, to control for indication bias, we assessed the relationship between the mode of delivery and maternal outcomes, while restricting the analysis to “low-risk” women, i.e. women aged <35 years, who had no medical conditions before or during the current pregnancy, no previous CS and who gave birth to a child weighting more than 2,500 grams (this criterion was used as a proxy for a term birth as we did not collect gestational age at delivery). Second, only early maternal deaths (within 24 hours after delivery) were included to take into account the fact that women with vaginal delivery tend to be discharged earlier compared to those with CS.

To take into account the hierarchical structure of the data, we used a hierarchical logistic mixed model with random intercept to model dependence of outcomes for individual women who delivered in the same hospital [13]. The effects of both individual and institutional factors on maternal and child outcomes were assumed to be the same for all hospitals and, accordingly, were modelled as fixed effects.

We calculated numbers needed to treat to benefit (NNTB) or to harm (NNTH) for maternal and child outcomes from the adjusted OR and its confidence interval [14].

All statistical analyses were performed using the SAS system software (SAS Institute Inc., Cary, NC, USA). Hierarchical logistic mixed models were estimated using the PROC NLMIXED procedure.

## Results

Among the 78,166 included women, 1,738 (2.2%) had a pre-labor CS and 76,428 (97.8%) had a trial of labor. Of them, 65,119 (85.2%) had a spontaneous vaginal delivery, 1,792 (2.3%) had an operative vaginal delivery and 9,517 (12.5%) had intrapartum CS. Maternal and fetal indications accounted for, respectively, 77.6% and 19.6% of all CSs (no indications reported in 2.8% of cases). The most commonly reported intrapartum CS indications were: prolonged/obstructed labor (44.6%), fetal distress (18.7%) and previous CS (12.3%) (Table 1). Previous CS (39.9%) and suspected cephalopelvic-disproportion (17.2%) were the most commonly reported pre-labor CS indications. In 18.5% of cases, pre-labor CSs were performed for “maternal indications” without any accurate diagnosis.

Women who underwent pre-labor CS were older, more likely to be multiparous, to have previous cesarean, medical history and pathologies during the current pregnancy (Table 2). As expected, compared to women who underwent spontaneous vaginal delivery, women who delivered by intrapartum CS were more likely to be referred from another hospital and to have obstetric complications such as pre-eclampsia and vaginal bleeding near full term. Overall, the proportion of low birth weight (LBW) was 15.5%. Women who delivered by pre-labor CS were more likely to have LBW babies, while the proportion of LBW was lower in women with operative vaginal delivery.

**Table 2:** Characteristics of women and newborns (n (%)), by mode of delivery (Senegal and Mali, October 2007-October 2008).

		Spontaneous vaginal delivery	Operative vaginal delivery	Intrapartum CS	Pre-labor CS
Women's characteristics					
	Age ≥35 years	8,904 (13.8)	148 (8.3)	1,216 (12.9)	439 (25.3)
	Nulliparous	22,332 (34.3)	1,097 (61.3)	4,292 (45.1)	444 (25.6)
	Previous cesarean section	2,179 (3.4)	123 (6.9)	2,074 (21.8)	902 (52.1)
	Any pathology before index pregnancy$	554 (0.9)	17 (1.0)	73 (0.8)	39 (2.2)
Current pregnancy					
	No prenatal visit	6,538 (10.1)	288 (16.3)	1,161 (12.3)	49 (2.9)
	Any pathology during current pregnancy£	2,227 (3.4)	64 (3.6)	213 (2.2)	99 (5.7)
	Pregnancy-induced hypertension	3,507 (5.4)	177 (9.9)	863 (9.1)	172 (9.9)
	Vaginal bleeding near full term	1,778 (2.7)	14 (0.8)	483 (5.1)	12 (0.7)
	Referred from another hospital	11,167 (17.2)	973 (54.3)	5,120 (54.8)	202 (11.6)
	Suspected intrauterine death	1,322 (2.0)	17 (1.0)	56 (0.6)	21 (1.2)
	Suspected intrauterine growth retardation	62 (0.1)	3 (0.2)	19 (0.2)	25 (1.4)
	Premature rupture of the membranes	1,910 (2.9)	37 (2.1)	602 (6.3)	17 (1.0)
	Premature labor	1,039 (1.6)	3 (0.2)	26 (0.3)	2 (0.1)
Newborns' characteristics¥					
	Sex (male)	30,949 (51.6)	927 (59.7)	4,816 (56.7)	872 (50.8)
	Mean (CI) birth weight (grams) (n)	2,925 (2,921–2,930) (n = 60,115)	2,958 (2,935–2,981) (n = 1,558)	3,061 (3,048–3,073) (n = 8,527)	2,992 (2,965–3,020) (n = 1,718)
	Low birth weight (<2,500 g)	7,735 (12.9)	131 (8.4)	897 (10.5)	246 (14.3)

CS, cesarean section.

$ HIV, chronic respiratory conditions, cardiac or renal diseases, sickle cell trait, chronic hypertension.

£ Pyelonephritis or urinary infection, chorioamniotis, severe maternal anaemia, malaria, gestational diabetes, suspected intrauterine death, suspected intrauterine growth retardation.

¥ Live singleton births only.

### Maternal and child outcomes

Maternal morbidity (ie., blood transfusion or hysterectomy) and vital status of the mother and the newborn were missing in 0.1%, 0.01% and 0.2% of cases, respectively.

A total of 388 (0.5%) women died during the labor or in the immediate post-partum period (Table 3). The most common reported causes of maternal death were post-partum haemorrhage (42.5%), hypertensive complications (19.9%) and indirect causes much represented by anaemia (20.7%) (Table 4). A total of 1,493 (1.9%) women had a blood transfusion or a hysterectomy.

**Table 3:** Specific maternal and child outcomes (n (%)), by mode of delivery (Senegal and Mali, October 2007-October 2008).

		All deliveries	Spontaneous vaginal delivery	Operative vaginal delivery	Intrapartum CS	Pre-labor CS
Maternal outcomes						
	Blood transfusion	1,402 (1.8)	909 (1.4)	28 (1.6)	448 (4.7)	17 (1.0)
	Hysterectomy	91 (0.1)	20 (0.03)	*n = *0	68 (0.7)	3 (0.2)
	Maternal death	388 (0.5)	209 (0.3)	22 (1.2)	155 (1.6)	2 (0.1)
Child outcomes^$^						
	Fetal/immediate neonatal death^&^	6,785 (8.7)	5,339 (8.2)	278 (15.6)	1,142 (12.1)	26 (1.5)
	Neonatal death after Day 1^£^	261 (0.4)	156 (0.3)	10 (0.7)	85 (1.0)	10 (0.6)

CS, cesarean section. ^$^Singleton births only.^ &^Stillbirth or neonatal death within 24 hours after birth occurred in, respectively, cases. ^£^Neonatal death that occurred between 24 hours after birth and hospital discharge.

**Table 4:** Causes of maternal death (n (%)), by mode of delivery (Senegal and Mali, October 2007-October 2008).

Cause of death	Spontaneous vaginal delivery	Operative vaginal delivery	Intrapartum CS	Pre-labor CS	All deliveries
Post-partum haemorrhage	94 (45.0)	8 (36.3)	61 (39.4)	2 (100.0)	165 (42.5)
Hypertensive complications	28 (13.4)	10 (45.4)	39 (25.2)	0	77 (19.9)
Obstructed labor	1 (0.5)	0	5 (3.2)	0	6 (1.5)
Puerperal infection	5 (2.4)	2 (9.1)	25 (16.1)	0	32 (8.3)
Rupture of the uterus	0	0	1 (0.6)	0	1 (0.2)
Other direct obstetric causes	14 (6.7)	1 (4.6)	9 (5.8)	0	24 (6.2)
Other indirect obstetric causes	64 (30.6)	1 (4.6)	15 (9.7)		80 (20.6)
Unknown	3 (1.4)	0	0	0	3 (0.8)
Total	209 (100.0)	22 (100.0)	155 (100.0)	2 (100.0)	388 (100.0)

CS, cesarean section.

A total of 6,132 (7.9%) stillbirths and 914 (1.3% of all live born babies) neonatal deaths were reported during the study period (Table 3). Among the 914 neonatal deaths, 71.4% (*n = *653) occurred during the first 24 hours after birth and 28.6% (*n = *261) between 24 hours after birth and hospital discharge. The child outcomes were 6,785/78,004 stillbirths and neonatal deaths within the first 24 hours after birth and 261/71,219 neonatal deaths after Day 1 in live born babies.

### Association between mode of delivery and maternal and child outcomes

After adjustment for individual risk factors and hospital characteristics, the risks of maternal mortality and morbidity associated with pre-labor CS did not differ significantly from those of women with a trial of labor (OR = 0.3 [0.07–1.32] and OR = 1.4 [0.86–2.28], for maternal mortality and maternal morbidity, respectively) (Table 5). Numbers needed to treat with pre-labor CS over one year were NNTB 286 (95% CI, NNTH 629 to ∞ to NNTB 215) and NNTH 139 (95% CI, NNTH 44 to ∞ to NNTB 393), for maternal mortality and morbidity respectively. Similar results were found when restricting the analyses to low-risk women (data not shown). Finally, we considered women with spontaneous vaginal delivery as the reference group. We did not find that maternal mortality differed significantly between women with spontaneous vaginal delivery and women with pre-labor CS (OR = 0.5 [0.10–1.96]), though maternal morbidity was significantly higher with pre-labor CS (OR = 2.1 [1.26–3.36]).

**Table 5:** Crude Odds ratio (ORc)* and adjusted Odds ratio (ORa)** of specific maternal and child outcomes in women with pre-labor cesarean section (CS) compared to women with a trial of labor (intention-to-treat analysis), and in women with operative vaginal delivery or intrapartum CS compared to women with spontaneous vaginal delivery (Senegal and Mali, October 2007-October 2008).

	Intention-to-treat		Trial of labor			
	Pre-labor CS *vs.* trial of labor		Operative vaginal delivery *vs.* spontaneous vaginal delivery		Intrapartum CS *vs.* spontaneous vaginal delivery	
	ORc (95% CI)	ORa (95% CI)	ORc (95% CI)	ORa (95% CI)	ORc (95% CI)	ORa (95% CI)
Maternal death$	0.3 (0.07–1.11)	0.3 (0.07–1.32)	3.3 (2.06–5.39)	2.5 (1.54–4.17)	4.5 (3.59–5.69)	3.2 (2.45–4.07)
Maternal transfusion and/or hysterectomy&	0.6 (0.39–0.97)	1.4 (0.86–2.28)	1.1 (0.75–1.67)	1.7 (1.14–2.66)	3.4 (2.99–3.83)	3.3 (2.83–3.86)
Fetal/immediate neonatal death£	0.2 (0.12–0.25)	0.2 (0.16–0.36)	1.9 (1.61–2.13)	2.2 (1.92–2.61)	1.3 (1.22–1.41)	1.1 (1.04–1.25)
Neonatal death after Day 1¥	1.3 (0.69–2.53)	1.3 (0.66–2.72)	3.8 (1.96–7.49)	5.9 (2.93–12.03)	3.9 (2.92–5.20)	4.7 (3.34–6.65)

Crude Odds ratio (ORc), Adjusted Odds ratio (ORa), confidence interval (CI).

* For each outcome of interest, the crude OR was corrected for the clustering effect of the facility and was adjusted for the time period (period 1: from October 2007 to March 2008; period 2: from April to October 2008) and the country.

** For each outcome of interest, the multivariable analysis was conducted using a hierarchical logistic mixed model with random intercept adjusted for individual and institutional variables (see the list below).

$ Adjustment for age, parity, previous CS, number of antenatal care visits, any pathology before index pregnancy, any pathology during current pregnancy, vaginal bleeding near full term, pregnancy-induced hypertension, chorioamniotis, referral status, availability of blood services, anaesthetist and obstetrics/gynaecology specialist availability, country and period.

& Adjustment for age, parity, number of antenatal care visits, previous CS, any pathology before index pregnancy, any pathology during current pregnancy, vaginal bleeding near full term, pregnancy-induced hypertension, chorioamniotis, referral status, country and period.

£ Fetal/immediate neonatal death consisted in stillbirth or neonatal death within 24 hours after birth. Adjustment for age, parity, number of antenatal care visits, previous CS, any pathology before index pregnancy, any pathology during current pregnancy, pregnancy-induced hypertension, vaginal bleeding near full term, chorioamniotis, premature rupture of membranes, birth weight, referral status, availability of formal protocols for maternal and neonatal care, anaesthetist and obstetrics/gynaecology specialist availability, country and period.

¥ Neonatal death after Day 1 consisted in neonatal death that occurred between 24 hours after birth and hospital discharge. Adjustment for age, parity, number of antenatal care visits, previous CS, any pathology before index pregnancy, any pathology during current pregnancy, pregnancy-induced hypertension, vaginal bleeding near full term, chorioamniotis, premature rupture of membranes, birth weight, referral status, availability of formal protocols for maternal and neonatal care, anaesthetist and obstetrics/gynaecology specialist availability, country and period.

Maternal mortality was higher in women with operative vaginal delivery (OR = 2.5 [1.54–4.17]) or intrapartum CS (OR = 3.2 [2.45–4.07]), compared with women who had spontaneous vaginal delivery (Table 5). The risk of maternal mortality did not differ significantly between women with intrapartum CS and those with operative vaginal delivery (OR = 1.2 [0.76–2.04]). While limiting the analysis to low-risk women, we replicated the significant increase of maternal mortality in the intrapartum CS and operative vaginal delivery groups compared to spontaneous vaginal delivery group (OR = 4.9 [2.55–9.26] and OR = 3.9 [1.34–11.47], respectively). When restricting the outcome to early maternal deaths (within the first 24 hours after delivery) only, the risk increases associated with both intrapartum CS (OR = 2.8 [2.08–3.86]) and operative vaginal delivery (OR = 2.5 [1.35–4.54]), relative to spontaneous vaginal delivery, remained statistically significant.

The risk of hysterectomy and/or blood transfusion adjusted on institutional and maternal characteristics was significantly increased in women who underwent intrapartum CS or operative vaginal delivery compared with women with spontaneous vaginal delivery (Table 5). Maternal morbidity was higher in women with intrapartum CS as compared to women with operative vaginal delivery (OR = 1.9 [1.25–2.93]). When limiting the analysis to low-risk women, we replicated a significant increased risk associated with intrapartum CS compared to spontaneous vaginal delivery (OR = 3.5 [2.88–5.21]), but operative vaginal delivery was only marginally associated with maternal morbidity (OR = 2.0 [0.97–4.29]).

After adjustment for maternal, perinatal and hospital characteristics, there was a marked reduction in the risk of fetal/immediate neonatal death in the group of women who delivered by pre-labor CS compared to women with a trial of labor (OR = 0.2 [0.16–0.36]) (Table 5). In contrast, the risk of neonatal mortality after Day 1 associated with pre-labor CS was not significantly different from that associated with a trial of labor (OR = 1.3 [0.66–2.72]). Numbers needed to treat with pre-labor CS over one year were NNTB 14 (95% CI, 14 to 28) and NNTH 927 (95% CI, NNTH 163 to ∞ to NNTB 816), for fetal/immediate neonatal mortality and neonatal mortality after Day 1 respectively. When considering spontaneous vaginal delivery as the reference group, we found similar results for fetal/immediate neonatal death (OR = 0.2 [0.16–0.38]), though neonatal death after Day 1 was significantly higher with pre-labor CS (OR = 2.0 [0.98–4.19]).

The risk of fetal/immediate neonatal mortality was two-fold higher with operative vaginal delivery compared to both spontaneous vaginal delivery (OR = 2.2 [1.92–2.61]) and intrapartum CS (OR = 2.0 [1.64–2.32]). Neonatal mortality after Day 1 was five and six-fold higher in intrapartum CS (OR = 4.7 [3.34–6.65]) and operative vaginal delivery (OR = 5.9 [2.93–12.03]) groups, respectively, compared with the spontaneous vaginal delivery group (Table 5). It did not differ between intrapartum CS and operative vaginal delivery (OR = 0.86 [0.39–1.61]).

## Discussion

### Principal findings

Our study represents one of the few studies that assessed the relation between mode of delivery and maternal and perinatal adverse outcomes in Africa using non-aggregate data.

We showed that the risks of in-hospital maternal mortality and neonatal mortality after Day 1 associated with pre-labor CS did not differ significantly from those of women with a trial of labor. Also, pre-labor CS was associated with a very important reduction in the risk of fetal/immediate neonatal death compared to women with a trial of labor. Among women undergoing a trial of labor, intrapartum CS and operative vaginal delivery were associated with two- to six-fold higher risks of maternal and neonatal mortality after Day 1 and maternal morbidity compared to spontaneous vaginal delivery, independent of women's, newborns' and hospitals' characteristics.

### Strengths and limitations

Several aspects of this study make our results of particular value. Firstly, we used data collected from most of the referral hospitals in Senegal and Mali. The 41 included hospitals were representative of the existing health system in both countries, taking into account the variety of contexts (urban *vs.* rural) and the levels of care. At the institutional-level, available equipment and human resources - which may have an impact on maternal and perinatal outcomes, were considered for analysis. All deliveries were prospectively recorded, with a proportion of included women among eligible women of 99%. Our findings may therefore be generalizable to other referral hospitals in sub-Saharan Africa with similar recruitments and characteristics. Given the large size of our study population, we could assess the associations between mode of delivery and the rare outcomes of maternal and neonatal deaths with sufficient statistical power (>80% in all analyses comparing intrapartum CS or operative vaginal delivery with spontaneous vaginal delivery). Secondly, we controlled for indication bias and, therefore, our results should better reflect the excess risk due to the procedures (CS or operative vaginal delivery) themselves rather than the clinical indications that led to the procedures. The main strategy used here was to perform an intention-to-treat analysis, which consisted in comparing maternal and child outcomes according to the antenatal decision about mode of delivery. We excluded very high risk women for CS (i.e., those with immediate life threatening maternal or fetal compromise), as we considered them as the most severe cases and, therefore, most likely to contribute to indication bias. Also, we adjusted for well known risk factors for maternal or perinatal poor outcomes, in accordance with publications from middle and low-income countries [1], [12], [15], [16]. This method allowed us to assess the excess maternal and neonatal mortality risks related to CS in all the patients except very high risk women, and to provide results which could be generalizable to all women delivering in referral hospitals in similar contexts without life threatening maternal and fetal compromise. Furthering this approach, we performed a sensitivity analysis including only low-risk women (i.e., with no identifiable medical risk factors) as proposed by authors from more developed countries [2], [3], [17], [18], and we obtained similar results. We did not have information available for congenital malformations and anomalies, which are known to be associated with poor perinatal outcomes. However, congenital malformations should not have been a confounding factor in this study as it is unlikely that they were detected prenatally – using an ultrasound scan – and that they affected the choice of delivery method. If residual confounding cannot be excluded, it is unlikely that it might explain the magnitude of the excess risks we estimated for CS and operative vaginal delivery compared to spontaneous vaginal delivery. In addition, the odds ratios we calculated were of the same level as those reported in other recent studies [1]–[3].

We closely monitored the accuracy of data on the vital status of the mother and the newborn at the facility-level and, therefore, misclassifications should have been minimised. However, some deaths that occurred after discharge from the hospital may have been missed. This may have affected both women with vaginal delivery, who are usually discharged shortly after delivery, and women with CS, who are at risk for dying due to late complications such as infection and venous thromboembolism. In total, misclassifications should not have been differential according to mode of delivery and, therefore, they should not have biased our results. A sensitivity analysis, in which only early maternal deaths were considered, did not change our results. Maternal morbidity was defined as blood transfusion or hysterectomy. One limitation of our study is that we did not include post-partum infection as an indicator of maternal morbidity, although it is one of the main causes of severe maternal morbidity associated with CS [17]. Indeed, length of follow-up was too short (36 hours after delivery in average) to allow the detection of post-partum infections, which are likely to occur later after delivery. For child outcomes, we used a composite variable combining fetal death and immediate neonatal death (i.e., within 24 hours after birth), as we could not distinguish between ante- and intrapartum stillbirths and misclassifications may have occurred between fetal and neonatal death.

### Discussion of results

The risk of maternal mortality associated with pre-labor CS was similar to that of women with a trial of labor. Although neonatal mortality after Day 1 was 30% higher with pre-labor CS than with intended vaginal delivery, the difference did not reach significance. This result differs from that of recent publications which have reported higher risks of neonatal mortality [1], [19]–[21] and severe adverse respiratory outcomes [19], [22] with pre-labor CS compared with intended vaginal delivery. One explanation may be that neonatal deaths due to respiratory complications after pre-labor CS have been underestimated, as women were discharged shortly after delivery and these complications are likely to occur more than 24 hours after birth. The power to detect a significant increased risk of neonatal death after pre-labor CS compared to trial of labour was 38% for a corresponding Odds ratio of 3.5 or more, as previously reported in term neonates [18]. Therefore, our results on neonatal mortality associated with pre-labor CS should be taken with caution. The risk of fetal/immediate neonatal mortality associated with pre-labor CS was lower compared with that of women with a trial of labor. This result is in agreement with previous publications [1] and it probably reflects the protective effect of pre-labor CS on the risk of labor-related complications (in particular, fetal asphyxia). Although we cannot exclude indication bias (i.e., pre-labor CS avoided in case of stillbirth), this should not explain the 80% decreased risk with pre-labor CS we observed. Also, it is unlikely that this protective effect was limited to breech pregnancies only [23], as they accounted for less than 5% in our study.

Together, these results highlight the relative safety of pre-labor CS to the relative risks associated with intrapartum CS and operative vaginal delivery. They suggest that in settings with similar health context pre-labor CS might be relevant in selected women at high risk for intrapartum intervention. However, the identification of such women is highly challenging in low-resource countries because indications for pre-labor CS such as cephalopelvic disproportion, malposition, abruption placentae or fetal distress are still poorly detected during antenatal care [24]. Also, both the identification of women who should benefit from a pre-labor CS and the decision to perform the intervention depends on the training and experience of the obstetrician. In a previous analysis conducted on the same databasis we showed that the presence of qualified medical staff (i.e., obstetrics specialists *vs.* trained general practitioners) was the main institutional predictor for pre-labor CS, independent of women and other institutional characteristics (Briand, personal communication). Interventions to improve the clinical decision-making for CS among health care professionals who offer antenatal services and among skilled birth attendants may contribute to higher pre-labor CS rates.

Compared to spontaneous vaginal delivery, intrapartum CS and operative vaginal delivery were associated with an increased risk of maternal and neonatal mortality. These findings are in agreement with those of publications from developed and developing countries [1], [2], [21] and add support for the recommendation of avoiding intrapartum interventions when there is not a clear medical indication that it will improve the outcome for the mother or the baby. In our study, the two main indications for intrapartum CS were dystocia and fetal distress, the diagnostic of which is as difficult as in high resource settings. The use of partograph – to identify obstructed labor – did not prove to reduce the risk of CS [25]. Also, the detection of fetal distress during labor using continuous cardiotocography has not been found to confer any clear benefit in terms of perinatal mortality compared to intermittent auscultation [26]. In low resource countries, the prevention of intrapartum related poor outcomes also depends on the ability to perform a timely CS. In our study, delays in transportation and in the decision to undertake intervention probably contributed to the increased morbidity and mortality associated with intrapartum CS and operative vaginal delivery. In such cases, interventions are performed too late to improve maternal and newborn outcomes.

### Conclusions and implications

Throughout West Africa, there is experimentation with fee exemption for cesarean and the effectiveness of this policy is of great importance. In view of the strengths and the limitations of our study, we showed that pre-labor CS is a safe procedure for the mother and the newborn. In contrast, intrapartum CS and operative vaginal delivery were associated with high risks of maternal and neonatal mortality. Emphasize may be given to interventions to improve the identification of women who may benefit from a pre-labor CS. Also, further research should be undertaken to determine what aspects of obstetric care contribute to a delay in the provision of intrapartum interventions so that practices may be made safer when they are needed.

## Supporting Information

Figure S1 **Flow chart of the study.** A total of 91,028 women delivered in the 46 referral hospitals during the study period. Five hospitals, in which 3,112 women delivered, were excluded from the analysis: four did not carry out any cesarean sections (CSs) during the study period and one only had data from mid-2008. Among the 87,916 women who delivered in the remaining 41 hospitals, 4,810 were excluded for one or more of the following reasons: place of living outside Senegal or Mali, spontaneous abortion (defined as birth weight less than 500 grams), multiple pregnancy, date or mode of delivery unknown. To limit confounding by indication, we also excluded 4,940 women with immediate life threatening maternal or fetal complication (severe pre-eclampsia, prerupture or rupture of the uterus, placenta praevia, transverse lie, brow presentation, or major cephalopelvic disproportion). Most (98%) of these women had a CS.
(TIFF)

## References

[1] Villar J, Carroli G, Zavaleta N, Donner A, Wojdyla D, et al (2007) Maternal and neonatal individual risks and benefits associated with cesarean delivery: multicentre prospective study. BMJ 335(7628): 1025. 17977819 10.1136/bmj.39363.706956.55 PMC2078636

[2] Deneux-Tharaux C, Carmona E, Bouvier-Colle MH, Breart G (2006) Postpartum maternal mortality and cesarean delivery. Obstet Gynecol 108(3 Pt 1): 541–548. 10.1097/01.AOG.0000233154.62729.24 16946213

[3] Kamilya G, Seal SL, Mukherji J, Bhattacharyya SK, Hazra A (2010) Maternal mortality and cesarean delivery: an analytical observational study. J Obstet Gynaecol Res 36(2): 248–253. 20492373 10.1111/j.1447-0756.2009.01125.x

[4] Clark SL, Belfort MA, Dildy GA, Herbst MA, Meyers JA, et al (2008) Maternal death in the 21st century: causes, prevention, and relationship to cesarean delivery. Am J Obstet Gynecol 199(1) 36: e1–5. 10.1016/j.ajog.2008.03.007 18455140

[5] Fournier P, Dumont A, Tourigny C, Dunkley G, Dramé S (2009) Improved access to comprehensive emergency obstetric care and its effect on institutional maternal mortality in rural Mali. Bull World Health Organ 87(1): 30–38. 19197402 10.2471/BLT.07.047076 PMC2649605

[6] Fenton PM, Whitty CJ, Reynolds F (2003) Cesarean section in Malawi: prospective study of early maternal and perinatal mortality. BMJ 327(7415): 587. 12969922 10.1136/bmj.327.7415.587 PMC194081

[7] Hussein J, Goodburn EA, Damisoni H, Lema V, Graham W (2001) Monitoring obstetric services: putting the ‘UN Guidelines’ into practice in Malawi: 3 years on. Int J Gynaecol Obstet 75(1): 63–73. 11597621 10.1016/s0020-7292(01)00474-x

[8] Stanton CK, Holtz SA (2006) Levels and trends in cesarean birth in the developing world. Stud Fam Plann 37(1): 41–48. 16570729 10.1111/j.1728-4465.2006.00082.x

[9] Dumont A, Fournier P, Fraser W, Haddad S, Traore M, et al (2009) QUARITE (quality of care, risk management and technology in obstetrics): a cluster-randomized trial of a multifaceted intervention to improve emergency obstetric care in Senegal and Mali. Trials 10: 85. 19765280 10.1186/1745-6215-10-85 PMC2758868

[10] Villar J, Valladares E, Wojdyla D, Zavaleta N, Carroli G, et al (2006) Cesarean delivery rates and pregnancy outcomes: the 2005 WHO global survey on maternal and perinatal health in Latin America. Lancet 367(9525): 1819–1829. 16753484 10.1016/S0140-6736(06)68704-7

[11] Ronsmans C, De Brouwere V, Dubourg D, Dieltiens G (2004) Measuring the need for life-saving obstetric surgery in developing countries. BJOG 111(10): 1027–1030. 15383102 10.1111/j.1471-0528.2004.00247.x

[12] Shah A, Fawole B, M'Imunya J M, Amokrane F, Nafiou I, et al (2009) Cesarean delivery outcomes from the WHO global survey on maternal and perinatal health in Africa. Int J Gynaecol Obstet 107(3): 191–197. 19782977 10.1016/j.ijgo.2009.08.013

[13] Molenberghs G, Verbeke G (2005) Models for Discrete Longitudinal Data. New York Springer.

[14] Altman DG (1998) Confidence intervals for the number needed to treat. BMJ 317; 1309. 10.1136/bmj.317.7168.1309 PMC1114210 9804726

[15] Lumbiganon P, Laopaiboon M, Gulmezoglu AM, Souza JP, Taneepanichskul S, et al (2010) Method of delivery and pregnancy outcomes in Asia: the WHO global survey on maternal and perinatal health 2007–08. Lancet 375(9713): 490–499. 20071021 10.1016/S0140-6736(09)61870-5

[16] Souza JP, Gülmezoglu A, Lumbiganon P, Laopaiboon M, Carroli G, et al (2010) Cesarean section without medical indications is associated with an increased risk of adverse short-term maternal outcomes: the 2004–2008 WHO Global Survey on Maternal and Perinatal Health. BMC Med 8: 71. 21067593 10.1186/1741-7015-8-71 PMC2993644

[17] Liu S, Liston RM, Joseph KS, Heaman M, Sauve R, et al (2007) Maternal mortality and severe morbidity associated with low-risk planned cesarean delivery versus planned vaginal delivery at term. CMAJ 176(4): 455–460. 17296957 10.1503/cmaj.060870 PMC1800583

[18] Allen VM, O'Connell CM, Liston RM, Baskett TF (2003) Maternal morbidity associated with cesarean delivery without labor compared with spontaneous onset of labor at term. Obstet Gynecol 102(3): 477–482. 12962927 10.1016/s0029-7844(03)00570-2

[19] De Luca R, Boulvain M, Irion O, Berner M, Pfister RE (2009) Incidence of early neonatal mortality and morbidity after late-preterm and term cesarean delivery. Pediatrics 123(6): e1064–1071. 19482739 10.1542/peds.2008-2407

[20] MacDorman MF, Declercq E, Menacker F, Malloy MH (2008) Neonatal mortality for primary cesarean and vaginal births to low-risk women: application of an “intention-to-treat” model. Birth 35(1): 3–8. 18307481 10.1111/j.1523-536X.2007.00205.x

[21] Gregory KD, Jackson S, Korst L, Fridman M (2012) Cesarean versus vaginal delivery: whose risks? Whose benefits? Am J Perinatol 29(1): 7–18. 21833896 10.1055/s-0031-1285829

[22] Hansen AK, Wisborg K, Uldbjerg N, Henriksen TB (2007) Pre-labor cesarean section and respiratory morbidity in the term and near-term neonate. Acta Obstet Gynecol Scand 86(4): 389–394. 17486457 10.1080/00016340601159256

[23] Hofmeyr GJ, Hannah ME (2003) Planned cesarean section for term breech delivery. Cochrane Database Syst Rev (3): CD000166. 10.1002/14651858.CD000166 12917886

[24] Campbell OM, Graham WJ (2006) Lancet Maternal Survival Series steering group (2006) Strategies for reducing maternal mortality: getting on with what works. Lancet 368(9543): 1284–1299. 17027735 10.1016/S0140-6736(06)69381-1

[25] Hofmeyr GJ, Haws RA, Bergström S, Lee AC, Okong P, et al.. (2009) Obstetric care in low-resource settings: what, who, and how to overcome challenges to scale up? Int J Gynaecol Obstet 107 Suppl 1: S21–44, S44–45. 10.1016/j.ijgo.2009.07.017 19815204

[26] Alfirevic Z, Devane D, Gyte GM (2006) Continuous cardiotocography (CTG) as a form of electronic fetal monitoring (EFM) for fetal assessment during labour. Cochrane Database Syst Rev (3): CD006066. 10.1002/14651858.CD006066 16856111

